# Traumatic Pancreatic Injury Presentation, Management, and Outcome: An Observational Retrospective Study From a Level 1 Trauma Centre in Northern India

**DOI:** 10.7759/cureus.90780

**Published:** 2025-08-22

**Authors:** Harsh V Baranwal, Vivek K Katiyar, Sumit Sharma, Vibhuti Uniyal, Shashi P Mishra, Satyanam K Bhartiya

**Affiliations:** 1 Department of General Surgery, Institute of Medical Sciences, Banaras Hindu University, Varanasi, IND; 2 Division of Trauma Surgery, Department of General Surgery, Institute of Medical Sciences, Banaras Hindu University, Varanasi, IND

**Keywords:** general trauma surgery, management of pancreatic injuries, pancreatic injury, retrospective observational study, traumatic pancreatic injury

## Abstract

Background

Pancreatic trauma (PT) is a rare but severe injury often associated with significant morbidity and mortality. Advances in diagnostic imaging and management strategies have led to a paradigm shift toward non-operative management (NOM) in select cases. This study aims to analyze the trends, outcomes, and challenges in the management of PT at a Level I trauma center over four years.

Methods

A retrospective observational study was conducted on 128 patients treated for PT between January 2021 and December 2024. Patients were categorized based on the American Association for the Surgery of Trauma (AAST) Pancreatic Injury Scale (Grades I-V). Data on demographics, injury characteristics, management strategies (operative vs. non-operative), complications, and outcomes were collected and analyzed. Statistical methods included chi-square tests, logistic regression, and descriptive analysis to evaluate predictors of outcomes and complications.

Results

Of the 128 patients, 78.9% were managed non-operatively, predominantly Grades I-II injuries, while 21.1% required surgical intervention, primarily for Grades III-V injuries. Complications were observed in 48.4% of patients, with pancreatic fistulas (21.9%) and sepsis (18.8%) being the most common. The mortality rate was 25.8%, with sepsis as the leading cause of death. NOM resulted in shorter hospital stays (median: 9 days, IQR: 7-14) compared to surgical management (median: 18 days, IQR: 12-25). Multivariate analysis identified high-grade injuries (AAST Grades IV-V) and sepsis as significant predictors of mortality.

Conclusion

The management of PT is increasingly leaning towards non-operative approaches for low-grade injuries, reflecting global trends. However, surgical intervention remains critical for high-grade injuries, especially those involving major ductal disruption or associated organ damage. This study emphasizes the importance of individualized care, early diagnosis, and multidisciplinary management to optimize outcomes.

## Introduction

Pancreatic trauma (PT) is an uncommon but potentially life-threatening condition, often encountered in the context of blunt or penetrating abdominal injuries. Despite advances in diagnostic modalities and management strategies, PT remains a challenging entity for trauma surgeons due to its anatomical location, probability of associated injuries to adjacent organs such as the liver, stomach, intestines, biliary system and major vessels [1]. The pancreas lies retroperitoneally, making its injuries difficult to detect on initial clinical evaluation. Moreover, the delayed onset of symptoms in low-grade pancreatic injuries often results in missed or delayed diagnoses, contributing to increased morbidity and mortality [2].

The rarity of PT is reflected in its reported incidence of less than 10% among abdominal injuries, with blunt trauma accounting for approximately 75% of cases and penetrating trauma for the remainder [3]. Common mechanisms of blunt injury include motor vehicle accidents and falls, while penetrating injuries typically result from gunshot or stab wounds. While PT is uncommon, its severity often correlates with the degree of injury to the pancreatic parenchyma and the involvement of the pancreatic duct, as classified by the American Association for the Surgery of Trauma (AAST) Pancreatic Injury Scale (Grades I-V) [4]. Injuries involving ductal disruption (Grades III-V) are associated with higher morbidity, particularly due to complications such as pancreatic fistulas, pseudocysts, abscesses, and pancreatic necrosis. The management of PT is further complicated by its association with polytrauma, as injuries to other abdominal organs, vascular structures, and the spine commonly coexist, necessitating a multidisciplinary approach to care [4,5].

Despite advancements, PT continues to be associated with significant morbidity and mortality, with sepsis and multi-organ failure (MOF) being the most common causes of death. Management complexities are magnified in low-resource settings where access to imaging and critical care may be limited. Even in tertiary care centers, the lack of standardized protocols for intermediate-grade injuries and the variability in long-term outcomes necessitate further research [5].

This study was undertaken to evaluate the presentation, grading, management strategies, and outcomes of PT at a Level I trauma center over a four-year period. By correlating clinical and surgical data with outcomes, we aim to assess the efficacy of non-operative management (NOM), identify predictors of complications and mortality, and contribute to the evolving understanding of PT management. During the study period (2021-2024), our institution applied the then-current AAST Pancreatic Injury Scale for grading. A revised version of the scale was published in 2025 (Notrica et al., 2025) [6], introducing refinements in ductal injury classification and descriptors for complex trauma. Although this update post-dates our data, it underscores the evolving framework for injury stratification and prognostication.

## Materials and methods

Study design

This study was a retrospective observational study conducted to analyze the presentation, management, and outcomes of pancreatic trauma. The design involved the evaluation of patient records, clinical details, and management protocols from a level I trauma center. The study relied on secondary data from electronic health records, operative notes, radiologic findings, and follow-up documentation. Data collection and analysis were designed to identify trends and outcomes associated with various management strategies based on injury severity. 

Study setting

The study was conducted at a Level I trauma center equipped with advanced diagnostic and therapeutic facilities, including a dedicated trauma surgery unit, intensive care units, and multidisciplinary care teams. This center served as a referral hub for high-complexity trauma cases, including pancreatic injuries, ensuring a comprehensive dataset for the study.

Study duration

All patients with PT admitted between January 2021 and December 2024 were included. The retrospective review and analysis were performed over February-June 2025, after the last patient’s discharge. Follow up, when available, extended for at least six months.

Objectives

The primary objective is to evaluate the presentation, management strategies, and outcomes of patients with PT at our Level I trauma center and the secondary objectives are (i) to assess complications, length of hospital stay, and mortality in relation to AAST injury grade, (ii) to identify predictors of adverse outcomes such as sepsis, and (iii) to analyze long term recovery based on management strategy.

Participants' inclusion and exclusion criteria

Participants included all patients diagnosed with PT during the study period. Inclusion criteria encompassed individuals of all ages and genders presenting with confirmed pancreatic injury (Grades I-V) as classified by the American Association for the Surgery of Trauma (AAST) Pancreatic Injury Scale. Patients were required to have complete medical records, including diagnostic imaging and management details. Exclusion criteria included patients with no in-hospital management data (e.g., direct transfer), incomplete records, or pre-existing pancreatic disease. Patients lost to outpatient follow-up after discharge remained in the analysis for inpatient outcomes.

Study sample size

A total of 128 patients met the inclusion criteria based on the total number of cases of pancreatic trauma treated at the center during the study period, representing a diverse cohort with varying grades of injury and associated management strategies.

Diagnostic evaluation

Initial tools on arrival were clinical exam and FAST. Definitive diagnosis/grading in stable patients was done using contrast-enhanced CT which was primary modality. MRI/MRCP and endoscopic retrograde cholangiopancreatography (ERCP) were selectively used as adjuncts for ductal injury when CT was equivocal. Sepsis was defined using Sepsis 3 criteria. Multi-organ failure (MOF) was defined as dysfunction in ≥2 organ systems requiring ICU level support.

Study procedure

The study began with a comprehensive review of the hospital’s medical record system to identify cases meeting the inclusion criteria. Patient records were reviewed for demographic details, mechanism and severity of injury, diagnostic imaging findings, and management strategies. Operative notes were examined for details of surgical interventions, including the type of procedure performed and intraoperative findings. For non-operative cases, records of supportive care, such as somatostatin analogs, nutritional support, and imaging follow-up, were analyzed. Data on complications, including sepsis, pancreatic leaks, and mortality, were extracted for outcome analysis. Data were collected using a standardized data collection sheet. Any ambiguities in the records were resolved through discussions with the treating surgeons or reviewing multidisciplinary case meeting notes.

Study parameters

The study parameters included demographic variables (age, gender), injury characteristics (mechanism of trauma, injury severity based on AAST grading), associated injuries, and management strategies. Outcomes were assessed based on the incidence of complications (e.g., fistulas, abscesses, pseudocysts), length of hospital stay, mortality, and the need for additional interventions during the hospital course.

Data analysis

The collected data were analyzed using IBM SPSS Statistics for Windows, Version 26 (Released 2019; IBM Corp., Armonk, New York, United States). Descriptive statistics were used to summarize demographic and clinical characteristics, while categorical variables were presented as frequencies and percentages. Chi-square or Fisher’s exact test was used for categorical variables. Student’s t-test or Mann-Whitney U test was used for continuous variables, depending on distribution. Predictors of mortality/complications were identified using multivariable logistic regression with odds ratios (OR) and 95% confidence intervals (CI). p < 0.05 was considered statistically significant.

Ethical considerations

As a retrospective observational study, the requirement for informed consent was waived. Patient confidentiality was strictly maintained by anonymizing all personal identifiers in the dataset. Data access was restricted to the study team, and all electronic records were stored on password-protected systems. The study adhered to the principles outlined in the Declaration of Helsinki and followed institutional and national guidelines for ethical research practices.

## Results

Admitting service and center volumes

Our Level I trauma center admits injured patients under four services: Trauma Surgery (torso trauma; major soft-tissue wounds; traumatic amputations; and extremity trauma not requiring orthopedic fixation), Orthopedics (skeletal injuries requiring fixation), Neurosurgery (traumatic brain/spine injury), and Plastic & Reconstructive Surgery (complex soft-tissue reconstruction and facial injuries). PT cases, irrespective of admitting service, were co-managed by Trauma Surgery and included if complete in-hospital pancreatic management data were available.

During the study period, the Trauma Surgery service admitted 2,796 patients; 1,226 (43.8%) had abdominal trauma. PT accounted for 128 admissions, representing 10.4% of abdominal trauma and 4.6% of all Trauma Surgery admissions.

Demographics and clinical characteristics

The study analyzed 128 patients with PT treated between January 2021 and December 2024. The mean age of the patients was 34.7 ± 10.2 years, with a male predominance (81.2%). The majority of injuries resulted from blunt trauma (77.3%), with motor vehicle accidents being the leading cause. Penetrating injuries accounted for 22.7%, primarily from stab wounds and gunshot injuries (Table 1).

**Table 1:** Demographic and Clinical Characteristics of the Study Population ^+^Vascular injury includes central vessels: portal vein, superior mesenteric vein, and splenic vessels, and peripheral vessels: segmental mesenteric branches and smaller intra-abdominal vessels.

Parameter	Value
Total Patients Admitted under Trauma Surgery	2796
Total Patients with Abdominal Trauma	1226
Total Patients with Pancreatic Injuries	128
Mean Age (years)	34.7 ± 10.2
Gender (Male:Female)	104:24
Mechanism of Injury	
- Blunt Trauma	99 (77.3%)
- Penetrating Trauma	29 (22.7%)
Associated Injuries	
Liver Injury	48 (37.5%)
Spleen Injury	36 (28.1%)
Thoracic Injury	30 (23.4%)
Vascular Injury^+^	29 (22.6%)
Bowel and Mesenteric Injury	26 (20.3%)
Renal Injury	18 (14.1%)
Pelvic Injury	11 (8.6%)
Diaphragmatic Injury	10 (7.8%)

Severity of pancreatic injury

The severity of PT was graded using the American Association for the Surgery of Trauma (AAST) scale. The majority of cases were classified as Grade I or II injuries (60.9%), while Grades III-V injuries accounted for 39.1%. Management strategies were categorized into non-operative and operative approaches. NOM was performed in 78.9% of cases, predominantly in patients with Grades I-II injuries. Surgical intervention was more common in Grades III-V injuries. With increasing injury severity, the proportion of patients requiring operative management rose markedly, reaching nearly 40% in Grade III, almost half in Grade IV, and 80% in Grade V injuries. This highlights the limited feasibility of NOM in high-grade injuries, reflecting the need for individualized decision-making based on hemodynamic stability and associated injuries (Table 2).

**Table 2:** Distribution of Pancreatic Injury Grades and Management Strategies Based on Injury Grade

AAST Grade	Patients n (%)	Non-operative Management n (%)	Operative Management n (%)
Grade I	35 (27.3%)	33 (94.3%)	2 (5.7%)
Grade II	43 (33.6%)	41 (95.3%)	2 (4.7%)
Grade III	28 (21.9%)	17 (60.7%)	11 (39.3%)
Grade IV	17 (13.3%)	9 (52.9%)	8 (47.1%)
Grade V	5 (3.9%)	1 (20.0%)	4 (80.0%)
Total	128 (100%)	101 (78.9%)	27 (21.1%)

Management strategies**

**Figure 1:**
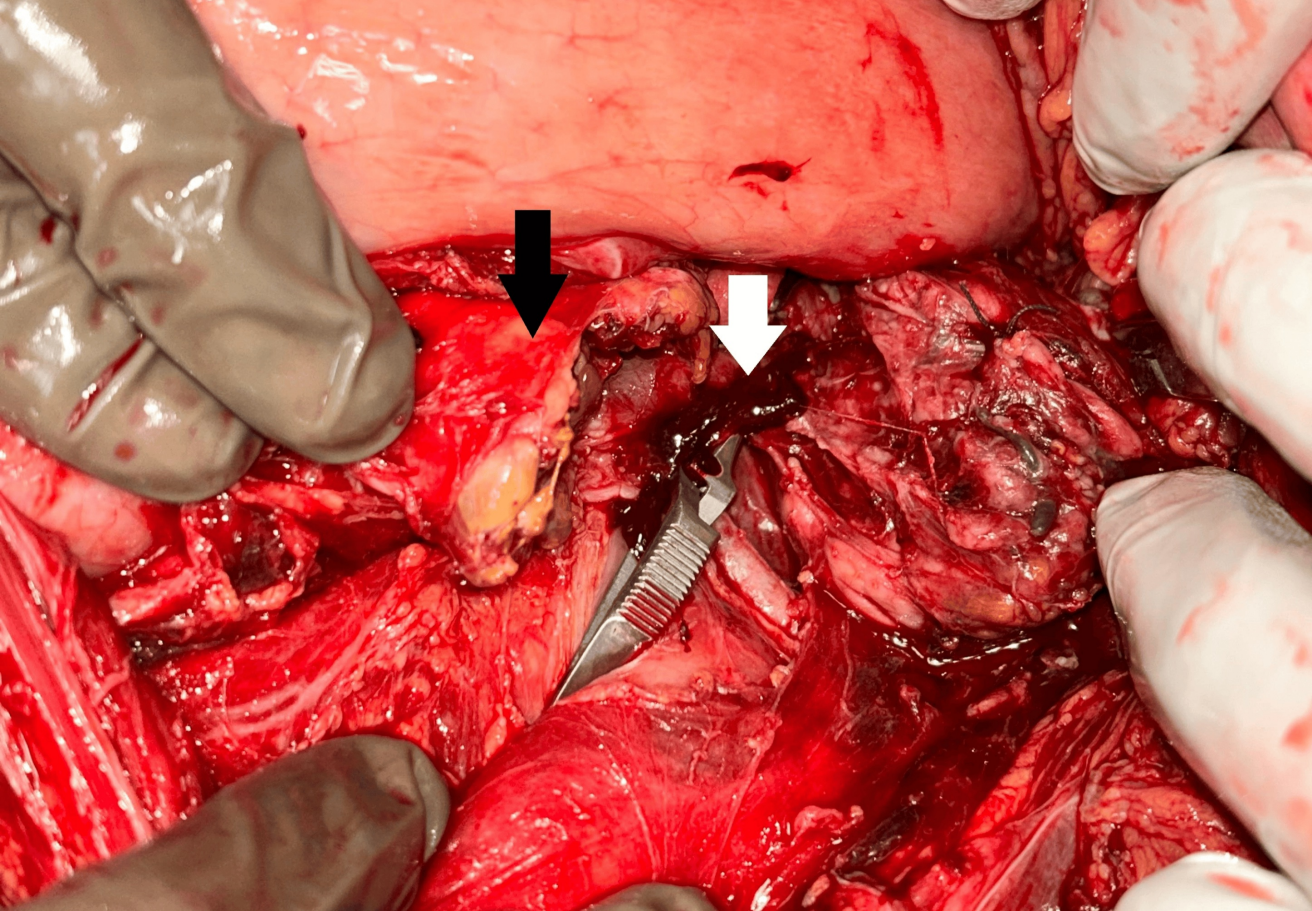
Grade 3 Pancreatic Injury (Transected Pancreatic Body: Black Arrow, Clamped Splenic Artery: White Arrow). The patient underwent distal pancreatectomy with splenectomy.

**Figure 2:**
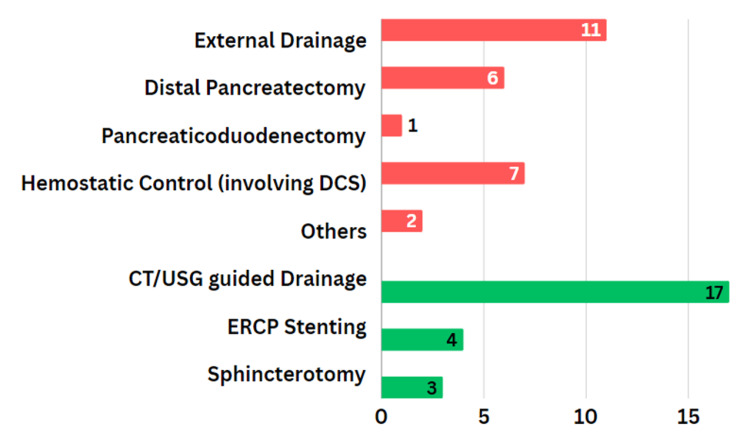
Graph Showing Distribution of Operative and Non-operative Procedures ERCP: Endoscopic retrograde cholangiopancreatography

The operative procedures varied based on the severity and location of the injury. Distal pancreatectomy was the most common procedure for injuries involving the pancreatic tail (Figure 1), while pancreaticoduodenectomy (Whipple procedure) and other procedures (debridement or partial resection) were performed in severe cases involving the pancreatic head. NOM was the initial strategy for most low-grade injuries (Grades I-II) and for selected hemodynamically stable patients with Grades III-IV without peritonitis or uncontrolled hemorrhage. Care included analgesia, early enteral nutrition, antibiotics when indicated, serial clinical examinations and labs, and imaging surveillance with CECT. When collections developed, CT/USG guided percutaneous drainage was performed (n=17), which constituted the most frequent NOM intervention. ERCP was used selectively in stable patients with suspected main pancreatic duct (MPD) injury on CT/MRCP or with persistent high output fistula. ERCP was done in seven patients of which pancreatic duct stenting was undertaken in four patients, and endoscopic sphincterotomy in three patients, to decompress the duct and facilitate resolution of leaks/collections, thereby helping to avoid or defer surgery in selected cases. ERCP availability was limited and performed in collaboration with the gastroenterology service; therefore, its use was not routine (Table 3; Figure 2).

**Table 3:** Distribution of Operative Procedures In our study, all distal pancreatectomies were performed with concomitant splenectomy, injuries involved significant splenic vessel disruption, associated splenic injury or hemodynamic instability.

Procedure	Number of Patients (%)
External Drainage	11 (40.7%)
Distal Pancreatectomy	6 (22.2%)
Pancreaticoduodenectomy	1 (3.7%)
Hemostatic Control Only	7 (25.9%)
Others	2 (7.4%)

Complications

The overall complication rate was 48.4% (62/128), with pancreatic fistulas and sepsis being the most common. The incidence and type of complications varied significantly across pancreatic injury grades. Complications were more frequent in patients with Grades III-V injuries (Table 4).

**Table 4:** Complications Observed in the Study Complications are presented according to the American Association for the Surgery of Trauma (AAST) pancreatic injury grading. Percentages represent the proportion of patients within each grade. ^†^Others include wound infection, pancreatic ascites and pleural effusion.

Complication	Grade I (n=35)	Grade II (n=43)	Grade III (n=28)	Grade IV (n=17)	Grade V (n=5)	Total (n=128)
Any Complication	3 (8.6%)	18 (41.9%)	20 (71.4%)	16 (94.1%)	5 (100.0%)	62 (48.4%)
Pancreatic Fistula	0 (0.0%)	5 (11.6%)	10 (35.7%)	11 (64.7%)	2 (40.0%)	28 (21.9%)
Sepsis	2 (5.7%)	3 (7.0%)	6 (21.4%)	9 (52.9%)	4 (80.0%)	24 (18.8%)
Pancreatic Abscess	1 (2.9%)	5 (11.6%)	4 (14.3%)	5 (29.4%)	1 (20.0%)	16 (12.5%)
Pseudocyst	0 (0.0%)	3 (7.0%)	5 (17.9%)	4 (23.5%)	0 (0.0%)	12 (9.4%)
Multi-organ Failure	0 (0.0%)	2 (4.7%)	2 (7.1%)	4 (23.5%)	3 (60.0%)	11 (8.6%)
Others^†^	0 (0.0%)	2 (4.7%)	2 (7.1%)	2 (11.8%)	2 (40.0%)	8 (6.3%)

Length of hospital stay

The median length of hospital stay (LOS) was 14 days (IQR: 9-21 days). The median LOS was notably longer in patients managed operatively compared with those managed non operatively, reflecting the increased severity of injuries and higher incidence of complications in surgically treated cases (Table 5).

**Table 5:** Length of Hospital Stay by Management Strategy LOS: Length of hospital stay. Values are expressed as median with interquartile range (IQR). Operative management included patients requiring laparotomy and pancreatic surgery, while non operative management comprised hemodynamically stable patients managed conservatively with or without endoscopic interventions.

Management Strategy	Median LOS (days)	IQR (days)
Non-operative	9	7–14
Operative	18	12–25
Overall	14	9–21

Mortality

Overall mortality was 25.8% across the cohort. Mortality increased sharply with injury severity and was highest in AAST Grades IV-V, irrespective of management strategy. In the NOM group, mortality was 18.0%, with deaths concentrated in Grades III-V. In the operative management (OM) group, mortality reached 55.6%, reflecting the greater physiological insult and injury burden in patients requiring surgery. No deaths occurred among OM patients with Grade I-II injuries, whereas a small number of NOM patients with low-grade injuries died, likely related to associated systemic factors or concomitant injuries rather than the pancreatic lesion itself. Notably, even among high-grade injuries (Grade III-IV), select patients managed non-operatively had comparatively lower mortality than those undergoing operative intervention, underscoring the importance of careful patient selection. Taken together, these data indicate that NOM is associated with lower mortality than OM, while high-grade injury remains the primary determinant of death regardless of treatment approach (Table 6).

**Table 6:** Mortality by Injury Grade Distribution of mortality stratified by AAST grade, comparing non-operative management (NOM) and operative management (OM). Percentages represent grade specific mortality within each treatment category.

AAST Grade	Total Patients	NOM (n)	OM (n)	Mortality NOM	Mortality OM	Total Mortality (%)
Grade I	35	33	2	1 (3.0%)	0 (0.0%)	1 (2.9%)
Grade II	43	41	2	4 (9.8%)	0 (0.0%)	4 (9.3%)
Grade III	28	17	11	6 (35.3%)	5 (45.5%)	11 (39.3%)
Grade IV	17	9	8	6 (66.7%)	6 (75.0%)	12 (70.6%)
Grade V	5	1	4	1 (100.0%)	4 (100.0%)	5 (100%)
Total	128	101	27	18 (18.0%)	15 (55.6%)	33 (25.8%)

Factors influencing mortality

On multivariate logistic regression analysis, several factors were found to be independently associated with mortality in PT. Higher AAST grade (IV-V), sepsis, and hemodynamic instability at presentation emerged as the strongest predictors, followed by low Glasgow Coma Scale (GCS <9) and the presence of associated injuries. The detailed results are presented in Table 7.

**Table 7:** Independent Predictors of Mortality in Pancreatic Trauma (Multivariate Logistic Regression Analysis) Multivariate logistic regression model showing predictors of mortality in pancreatic trauma. Higher AAST grade, presence of sepsis, hemodynamic instability, low GCS, and associated injuries were found to be significant predictors. Hemodynamic instability at presentation: SBP <90 mmHg GCS: Glasgow Coma Scale

Variable	Odds Ratio (95% CI)	p-value
AAST Grade (IV–V)	4.5 (2.1–9.8)	0.001
Sepsis	3.8 (1.7–8.6)	0.002
Hemodynamic instability at presentation	3.9 (1.6–9.4)	0.003
GCS <9	3.0 (1.19–7.9)	0.035
Associated injuries	2.2 (1.1–4.3)	0.031

Long-term outcomes

Follow-up data were available for 72 out of 95 surviving patients (75.79%). Among these, 77.5% of patients managed non-operatively had complete recovery, while 53.1% of those managed operatively reported ongoing symptoms, such as abdominal pain or impaired exocrine function (Table 8).

**Table 8:** Long-Term Outcomes at Six-Month Follow-Up Values are given as n (% of total survivors, n=72). Patients may have multiple overlapping symptoms; therefore, symptom counts exceed totals. Exocrine insufficiency included steatorrhea and malabsorption. FE-1 <200 µg/g stool or (if unavailable) steatorrhea with ≥5% weight loss and improvement on PERT. Endocrine dysfunction included new-onset diabetes/poor glycemic control. ADA criteria for new-onset diabetes or escalation of therapy for pre-existing diabetes. Assessed at one, three, and six months. PERT: Pancreatic Enzyme Replacement Therapy; ADA: American Diabetes Association

Outcome	n	% (of 72)
Complete Recovery	46	63.90%
Persistent Symptoms	26	36.10%
Chronic abdominal pain	13	18.10%
Exocrine insufficiency	8	11.10%
Endocrine dysfunction	6	8.30%
Recurrent pancreatitis	4	5.60%
Other complications	5	6.90%

## Discussion

Historically, PT management centered on laparotomy for diagnosis and repair, using debridement, external drainage, and when necessary resection or pancreatectomy [7]. With advances in CT/MRI and a clearer understanding of the natural history of low-grade injuries, practice has shifted toward selective NOM in hemodynamically stable patients without ductal or major vascular involvement [8,9]. For Grades I-II, conservative care with close monitoring and serial imaging is often sufficient; typical measures include bowel rest, nutritional support, somatostatin analogs, and prophylactic antibiotics, with vigilance for pseudocyst, abscess, or secondary infection that may prompt delayed intervention [10-12].

By contrast, higher-grade injuries (III-V), specially those with main duct disruption, generally warrant operative management to control leakage and prevent severe inflammatory sequelae; procedure choice depends on injury location (e.g., distal pancreatectomy for tail, pancreaticoduodenectomy for complex head injuries), and damage control surgery is reserved for the unstable patient as a staged approach [13-15].

The global shift towards NOM for select cases of PT has paralleled advancements in trauma care systems, particularly in high-resource settings. Level I trauma centers equipped with state-of-the-art imaging facilities, multidisciplinary teams, and intensive care units have facilitated early and accurate diagnosis, enabling clinicians to stratify patients based on injury severity and individualize treatment plans [16]. Furthermore, the integration of endoscopic and interventional radiologic techniques has expanded the therapeutic options available for pancreatic injuries. ERCP has emerged as a valuable tool for both diagnostic and therapeutic purposes, allowing for the identification of ductal injuries and the placement of stents to promote ductal healing or manage leaks [17]. Similarly, percutaneous drainage of collections under radiologic guidance has reduced the need for invasive surgical procedures in cases of infected pancreatic pseudocysts or abscesses [18].

The broader adoption of NOM has paralleled improvements in trauma systems and imaging access [16], while ERCP and interventional radiology have expanded diagnostic and therapeutic options for ductal injury and septic collections [17,18]. Persistent challenges include delayed diagnosis and limited advanced modalities in low-resource settings [19], as well as ongoing debates regarding Grade III management, timing of surgery, antibiotic prophylaxis, and long-term outcomes [20].

The morbidity and mortality associated with PT remain high, with reported mortality rates ranging from 15% to 45%, depending on the severity of the injury and the presence of associated complications [21]. Sepsis and MOF are the leading causes of death in these patients, underscoring the importance of timely intervention and aggressive management of complications. Moreover, the psychological and economic burden of PT on patients and healthcare systems is substantial, further emphasizing the need for evidence-based approaches to optimize outcomes [22].

Morbidity and mortality remain substantial (≈15-45%), driven largely by sepsis and MOF, with significant psychosocial and economic burden [21,22]. A multidisciplinary, protocolized approach including modern resuscitation strategies and quality improvement frameworks continues to be emphasized to improve survival and reduce complications [23,24].

This analysis was conducted at a tertiary, academic Level I trauma center with a high patient throughput and round-the-clock multispecialty support (general/HPB surgery, orthopedics, neurosurgery, urology, anesthesia, and critical care). Contrast-enhanced CT is available continuously, MRCP is obtained selectively, and therapeutic ERCP is not routinely accessible, which is pertinent when interpreting duct-focused nonoperative pathways. The study cohort reflects the regional trauma demography: predominantly young to middle-aged males, with blunt mechanisms (road-traffic collisions and falls) forming the majority and penetrating injuries comprising a minority. Polytrauma with associated thoraco-abdominal and extra pancreatic injuries was common. These contextual factors: case-mix, resource availability, and referral patterns, frame the management choices observed between January 2021 and December 2024 and inform interpretation of outcomes across grades and operative and nonoperative strategies.

The demographic characteristics observed in this study align with findings in previous research studies. Male predominance (81.2%) with a mean age of 34.7 years is consistent with the global epidemiological pattern of PT, as reported by Scollay et al. (2006), who documented a 3:1 male-to-female ratio with a median age of 32 years in their population-based study in Scotland [25]. Similarly, O’Reilly et al. (2015) [26] found a male-to-female ratio of 2.5:1 and a median age of 27 years for patients with pancreatic injuries in the UK, emphasizing the similarity in demographic patterns across different regions. Blunt trauma accounted for 77.3% of injuries in this study, predominantly caused by motor vehicle accidents, paralleling findings by Scollay et al. (2006), who reported 66% of injuries as blunt trauma, with road traffic accidents being the leading cause. Additionally, penetrating injuries constituted 22.7% of cases, reflective of urban trauma patterns, a proportion similar to that found by Phillips et al. (2018) [27] in their analysis of penetrating pancreatic injuries in the National Trauma Data Bank, where penetrating trauma comprised a significant portion of cases. The high prevalence of associated injuries, such as those to the liver (37.5%) and spleen (28.1%), also aligns with the findings of O’Reilly et al. (2015) [26], who noted that most pancreatic injuries occurred alongside other abdominal trauma, with liver and splenic injuries being common. This underscores the complexity of managing PT, given the high rate of concurrent injuries.

The distribution of injury severity in this study aligns with patterns reported in previous research, emphasizing the variability in PT severity and its management. The finding that 60.9% of cases were classified as low-grade injuries (Grades I-II) is consistent with Scollay et al. (2006), who reported that low-grade injuries (Grade I-II) comprised the majority of PT cases in their population-based study, accounting for approximately 60% of cases. Similarly, O’Reilly et al. (2015) [26] found that low-grade injuries were common, highlighting their frequent association with non-operative management approaches. The trend toward NOM for low-grade injuries observed in this study, with 95.3% of Grade II injuries managed non-operatively, mirrors findings from O’Reilly et al. (2015) [26], who reported that hemodynamically stable patients with low-grade injuries were predominantly managed conservatively. This approach has been supported by evolving guidelines prioritizing NOM for stable patients with Grades I-II injuries. For high-grade injuries (Grades III-V), the need for surgical intervention in 39.3% Grade III, 47.1% Grade IV, and 80.0% Grade V injuries in this study is comparable to findings by Phillips et al. (2018) [27], who reported that operative management was predominantly required for high-grade injuries due to complications such as ductal disruption and tissue damage. Their analysis showed that Grade V injuries nearly always necessitated surgical intervention, reflecting their critical nature.

Complications increased stepwise with AAST grade (Any complication: Grade I 8.6%, Grade II 41.9%, Grade III 71.4%, Grade IV 94.1%, Grade V 100%; Table 6). Higher-grade injuries were associated with greater rates of fistula, sepsis, abscess, and MOF. Unadjusted comparisons favored NOM over OM. Because operative patients disproportionately had higher grade injuries, these differences are susceptible to selection (grade/severity) bias and should be interpreted alongside the grade-stratified results. The overall complication rate of 48.4% in this study aligns with findings in prior research, emphasizing the significant morbidity associated with PT, particularly in operatively managed cases. Akhrass et al. (1997) [28] reported a similar overall complication rate of 42%, with pancreatic fistulas (11%) and pancreatitis (7%) being among the most frequent complications. Their findings also highlighted higher morbidity rates in operatively managed patients, particularly those with low-grade injuries subjected to unnecessary drainage, underscoring the risks associated with surgical interventions. Similarly, Gaspar et al. (2021) [29] documented high complication rates in surgically treated PT cases, with an 80% complication rate for high-grade injuries requiring operative management. This study also emphasized the increasing trend towards non-operative management for stable patients, reporting shorter hospital stays and fewer complications in non-operatively managed cases. The need for careful monitoring in NOM to address complications such as pseudocysts and abscesses was also noted, mirroring the findings of this study. These comparisons reinforce the understanding that while operative management is often necessary for high-grade injuries, it carries significant risks of complications and prolonged hospital stays. Conversely, NOM offers advantages in selected cases but requires vigilant monitoring to address delayed complications effectively.

NOM predominated overall (78.9%, 101/128), especially in low-grade injuries, with 94.3% of Grade I and 95.3% of Grade II cases managed non-operatively. The need for surgery increased with injury severity: 39.3% in Grade III, 47.1% in Grade IV, and 80.0% in Grade V. Distal pancreatectomy (all with splenectomy) was the most common resection, pancreaticoduodenectomy was rare (1/27 operative cases; 3.7%; 0.8% of the cohort), and drainage procedures (surgical or image-guided) were the most frequent interventions (Figure 1; Tables 3-4). Operative interventions in this study showed a pattern consistent with established surgical practices for PT. This aligns with observations by Biffl et al. (2022) [9], who reported this procedure as critical for managing high-grade proximal injuries while noting its association with significant morbidity. External drainage, performed in 40.7% of cases, was primarily utilized to manage pancreatic leaks and localized infections, consistent with Gaspar et al. (2021) [29], who emphasized its role in addressing postoperative complications while minimizing additional surgical risks. These findings reinforce that the choice of surgical intervention remains heavily dependent on the anatomical location and severity of the injury, as well as the patient's clinical condition.

Overall mortality was 25.8% (33/128) and rose sharply with injury severity, reaching 70.6% in Grade IV and 100% in Grade V (Tables 6, 7). By management strategy, mortality was 18.0% in NOM (18/101) and 55.6% in OM (15/27), reflecting the injury burden among surgical candidates. No deaths occurred among OM patients with Grade I-II injuries, whereas a small number of NOM patients with low-grade injuries died, likely related to associated systemic factors rather than the pancreatic lesion itself. A small DCS subgroup (n=6) presented with profound instability; early deaths prior to sepsis onset influenced overall patterns. In their study, O’Reilly et al. (2015) [26] reported a mortality rate of 17.6% for blunt pancreatoduodenal injuries and 12.2% for penetrating injuries, with a significant increase in mortality linked to higher injury severity and associated complications. Similarly, Scollay et al. (2006) highlighted a mortality rate of 46% in their population-based study, noting that deaths were more common in patients with Grade IV-V injuries, hemodynamic instability, and polytrauma.

The identification of sepsis (OR 3.8) as a significant predictor of mortality is consistent with findings by Gaspar et al. (2021) [29], who reported that sepsis and MOF were leading causes of death in patients with high-grade pancreatic injuries. The strong association between vascular injuries and increased mortality (OR 2.2) is corroborated by Phillips et al. (2018) [27], who demonstrated that major vascular involvement significantly exacerbates outcomes due to the complexity of surgical management and risk of hemorrhagic shock. In our study, on multivariable analysis, independent predictors of mortality were AAST Grade IV-V (OR 4.5, 95% CI 2.1-9.8; p=0.001), sepsis (OR 3.8, 1.7-8.6; p=0.002), hemodynamic instability at presentation (SBP <90 mmHg) (OR 3.9, 1.6-9.4; p=0.003), GCS <9 (OR 3.0, 1.19-7.9; p=0.035), and associated injuries (OR 2.2, 1.1-4.3; p=0.031) (Table 7). These findings underscore the critical importance of injury severity and the presence of complications like sepsis and vascular damage in shaping mortality outcomes, highlighting the need for aggressive monitoring and timely intervention in high-risk patients.

The long-term follow-up data in this study further highlight the morbidity associated with PT. This aligns with Gaspar et al. (2021) [29], who reported better long-term outcomes and lower rates of chronic complications such as pain or pancreatic insufficiency among patients managed conservatively, particularly for low-grade injuries. Follow-up was available in 72 patients (75.8%). Median LOS was longer after surgery, 18 days (IQR 12-25) vs 9 days (IQR 7-14) for NOM, consistent with higher injury severity and complication burden. Because injury grade strongly influenced treatment allocation, comparisons between NOM and OM are prone to selection bias; accordingly, interpretation prioritizes grade-specific outcomes and multivariable results. Complete recovery was more frequent after NOM (77.5%), whereas 53.1% of OM patients reported persistent symptoms. Among survivors, chronic abdominal pain (18.1%) and exocrine insufficiency (11.1%) were most common. These findings underscore the advantages of NOM in selected patients, where the avoidance of surgical complications and the reduced risk of long-term morbidity are evident. However, the challenges faced by patients requiring complex surgeries, particularly those with high-grade injuries, underline the need for prolonged rehabilitation, multidisciplinary care, and vigilant follow-up to address chronic complications effectively. This further reinforces the importance of imaging-based management strategies based on injury grade, patient stability, and long-term recovery potential.

Placing our findings alongside contemporary cohorts (Table 9) highlights three consistent signals: first, reported operative rates vary widely (≈35-80%) largely reflecting differences in case mix, referral patterns, and how studies define NOM (observation alone vs inclusion of ERCP/IR); second, overall mortality tracks far more with injury severity and associated trauma than with management strategy per se, with large datasets that are skewed to low grade injuries showing low mortality despite high NOM use [3], while single center series enriched for high-grade lesions report higher mortality [1]; and third, the utility of endoscopic/radiologic adjuncts appears to expand the safe envelope of NOM for AAST I-III injuries but has limited impact once AAST IV-V ductal disruption and polytrauma physiology dominate [30]. In this context, our center’s overall mortality of 25.8% sits within the range reported by high-grade, single-center experiences and is notably concentrated in AAST IV-V injuries irrespective of management, underscoring that grade and physiologic derangement, not the choice of operative vs non-operative pathway, are the principal drivers of death. These comparisons support a pragmatic approach: prefer NOM (with early ductal assessment and selective ERCP/IR) for low-grade injuries, while prioritizing timely operative control/resection or drainage for high-grade lesions within a damage control framework, with outcomes best improved by optimizing resuscitation and addressing extra pancreatic injuries.

**Table 9:** Operative vs Non-operative Management and Mortality across Contemporary Pancreatic Trauma Cohorts ^†^Overall mortality for Addison derived from the reported group rates and denominators (8/29 + 1/32 ≈ 9/61). Source reports the group-specific rates explicitly. ^‡^Many trauma series classify ERCP/IR as non-operative management (NOM); Meijer reports them separately. If we take “no-laparotomy = NOM” definition here, NOM would be 40 (conservative) + 16 (ERCP/IR) = 56/165 (33.9%) vs. operative 106/165 (64.2%). NOM is recorded as defined by each study; endoscopic/radiologic interventions (e.g., ERCP, percutaneous drainage/angiography) are counted within NOM when so defined in the source. Heterogeneity in era, injury grade mix, and associated injuries limits direct cross study comparisons; figures are provided to contextualize our center’s outcomes. ERCP: Endoscopic retrograde cholangiopancreatography; IR: interventional radiology

Study (Year, Setting)	N (patients)	Operated (n / %)	Non-operative (n / %)	Mortality (Overall)	Mortality by Strategy (If Reported)
Addison 2016, multi-institutional (Burns & Trauma) [4]	61	29 / 47.5%	32 / 52.5%	~14.8%^†^	Operative 27.6% vs NOM 3.1%.
Al-Thani 2022, Qatar (Frontiers in Surgery) [1]	71	37 / 52.1%	32 / 45.1%	31.00%	Not Broken Down by Strategy.
Meijer 2023, Europe Multicenter (Heliyon) [3]	165	106 / 64.2%	40 / 24.2% “Conservative”; +16 / 9.7% ERCP/IR^‡^	6.70%	Not Broken Down by Strategy.
Khalayleh 2022, Israel Single-Center [30]	77	47 / 61.0%	30 / 39.0% (14 Failures → op)	10.40%	0% in Successful NOM; All Deaths (8/77) in the operative group.
Gupta 2016, AIIMS (India) Level-1 [23]	53	43 / 81.1%	10 / 18.9%	20.8% (11/53)	Operative 23.3% (10/43) vs NOM 10.0% (1/10).
This Study	128	27 / 21.1%	101 / 78.9%	25.8% (33/128)	Operative 55.6% vs NOM 18.0%

The increasing adoption of NOM has been facilitated by advancements in imaging techniques, which allow for accurate injury grading and monitoring. Computed tomography (CT) and magnetic resonance imaging (MRI) played a pivotal role in the early diagnosis and follow-up of patients in this study, enabling timely intervention for complications such as pseudocysts and abscesses. The findings also highlight the need for a multidisciplinary approach to PT management. Collaboration between trauma surgeons, radiologists, gastroenterologists, and critical care specialists is essential for optimizing patient outcomes. ERCP was used selectively, principally for suspected MPD injury or persistent high output leaks, with pancreatic duct stenting in four patients and endoscopic sphincterotomy in three patients; ERCP availability was limited and not routine (Figure 1; Results). Similarly, percutaneous drainage of abscesses and collections reduced the need for repeat laparotomies, reflecting the evolving role of interventional radiology in trauma care.

Despite these advancements, challenges remain in the management of PT. The high complication and mortality rates observed in this study highlight the need for ongoing research to refine existing management protocols. Additionally, the development of standardized follow up protocols could help identify and address long-term complications more effectively.

Limitations

This study has several limitations. First, its retrospective and single-center design limits the generalizability of the findings. Second, a standardized trauma severity score such as the Injury Severity Score (ISS) was not applied, making it difficult to distinguish outcomes attributable solely to PT versus polytrauma. Third, the comparison of operative and non-operative groups may be biased, as higher-grade injuries were disproportionately represented in the operative cohort. Fourth, advanced imaging modalities such as MRI and ERCP were not uniformly available, which may have influenced diagnostic accuracy and subsequent management decisions. Finally, cost-effectiveness analysis and detailed resource utilization were beyond the scope of this study.

## Conclusions

This study reinforces that NOM is safe and effective in hemodynamically stable patients with low-grade pancreatic injuries (AAST Grade I-II), while high-grade injuries carry very high mortality (70% in Grade IV, 100% in Grade V). These findings highlight the critical need for early recognition of injury severity and tailored management strategies. NOM should be reserved for carefully selected stable patients with low-grade trauma, whereas high-grade injuries demand timely surgical intervention and aggressive critical care support. Sepsis emerged as a major determinant of poor outcomes, emphasizing the importance of close monitoring and early detection. A multidisciplinary approach incorporating surgeons, intensivists, radiologists, and gastroenterologists is essential for optimizing patient outcomes in this challenging cohort. The retrospective, single-center design and limited availability of advanced imaging modalities such as MRI and ERCP may have influenced diagnostic accuracy and subsequent management decisions, restricting generalizability. Nonetheless, the data provide actionable implications: stable, low-grade injuries should be managed conservatively; high-grade injuries require aggressive management; and sepsis surveillance should be integrated as a routine component of care. Future research should focus on addressing the unresolved challenges in PT management to further improve outcomes for this complex and life-threatening condition.
